# Evaluation of Pulse Oximetry in the Early Detection of Cyanotic Congenital Heart Disease in Newborns

**Published:** 2016-04-13

**Authors:** Amir Hosein Movahedian, Ziba Mosayebi, Setareh Sagheb

**Affiliations:** *Tehran University of Medical Sciences, Tehran, Iran.*

**Keywords:** *Congenital- hereditary- and neonatal diseases and abnormalities*, *Heart defects- congenital*, *Infant- new born*, *Oximetry*

## Abstract

**Background:** Delayed or missed diagnosis of critical and cyanotic congenital heart disease (CHD) in asymptomatic newborns may result in significant morbidity and mortality. The aim of this study was to determine the accuracy of pulse oximetry screening performed on the first day of life for the early detection of critical and cyanotic CHD in apparently normal newborns.

**Methods:** This cross-sectional study used postductal pulse oximetry to evaluate term neonates born between 2008 and 2011 with normal physical examinations. Functional oxygen saturation < 95% was considered abnormal, and second measurement was done 2 hours later. If the second measurement remained < 95%, an echocardiogram was performed. On enrolment in the study, the following data for each neonate were recorded: gestational age, gender, birth weight, mode of delivery, and mother’s age.

**Results: **During the study period, totally 3,846 newborns were evaluated. Of the whole study population, 304 (7.9%) babies had a postductal functional saturation < 95%. The second measurement was also < 95% in 104 (2.7%) neonates. The mean age of the neonates at the time of pulse oximetry was 18.91 ± 8.61 (min = 4.5 and max = 49) hours. Forty-nine percent of the subjects were female and 51% were male. Echocardiography was performed on 81 out of 104 newborns, and 14 (0.36%) of them had CHD. The types of CHD in our patients were tetralogy of Fallot (3 cases), transposition of the great vessels (2 cases), double-outlet right ventricle (2 cases), truncus arteriosus, total anomalous pulmonary venous return, atrioventricular septal defect, pulmonary atresia, persistent pulmonary hypertension, ventricular septal defect, and atrial septal defect (1 case for each type). The best time for pulse oximetry was within 8-24 hours of the newborns’ life.

**Conclusion: **Pulse oximetry screening along with clinical examination may be able to assist in the early detection of critical and cyanotic CHD in asymptomatic newborns.

## Introduction

Congenital heart disease (CHD) is the most common congenital malformation, with an incidence of 8 per 1000 live births. Critical CHD, defined as CHD requiring early detection and surgical intervention or cardiac catheterization in the first year of life to sustain life, occurs in 2.5 to 3 per 1,000 live births.^1^ These cardiac lesions are responsible for approximately 40% of deaths due to congenital birth defects in the first year of life.^2^ Prenatal ultrasound can detect < 50% of the cases of critical CHD. Signs and symptoms suggestive of CHD are not always present initially in the first few days of life, so physical examination does not always recognize neonates with CHD. Despite prenatal ultrasound and postnatal clinical examinations, up to 39% of infants with critical CHD will leave the hospital before a diagnosis could be made^3^ and about 43% of them will return to the hospital in hemodynamic instability or shock status and some may die at home without any diagnosis.^4^ Therefore, early diagnosis of critical CHD is important because delayed diagnosis could be associated with sudden deterioration, cardiovascular compromise, end organ damage, and even death.^5^

Pulse oximetry is a simple, fast, inexpensive, and noninvasive method which can be used to show oxygen saturation in blood. As there are some scales of hypoxia in severe cyanotic heart disease not detectable by the patient’s appearance, pulse oximetry could be a great advancement in this regard.

Numerous studies have revealed the value of pulse oximetry in the screening of CHD in newborns before discharge.^6^^-^^8^ Hoke et al.^9^ were able to detect hypoplastic left-heart syndrome, coarctation of the aorta, and tetralogy of Fallot using concomitant pulse oximetry in both hand and foot in neonates without symptoms. 

In 2011, the American Academy of Pediatrics (AAP) and the American Heart Association (AHA) endorsed pulse oximetry screening for the early detection of critical CHD to prevent subsequent morbidity and mortality that may occur as a result of late diagnosis.^10^

To the best of our knowledge, there are no data on the use of pulse oximetry as a screening test for the diagnosis of critical CHD in neonates in Iran. The aim of this study was to evaluate the role of pulse oximetry in the early detection of asymptomatic critical CHD in newborns before discharge from nursery.

## Methods

This cross-sectional study, conducted from 2008 to 2011, evaluated term neonates who were normal according to standard neonatal examinations. The study protocol was approved by the Research Ethics Committee of Kashan University of Medical Sciences. Informed consent was obtained from the parents. The newborns were enrolled in the study by simple sampling, and pulse oximetry was done on their right foot. Newborns with a gestational age < 37 weeks, sick babies admitted to neonatal units, and those whose parents refused consent were excluded from the survey. Pulse oximetry was performed for all the healthy term newborns at least 2 hours after birth by the well-experienced personnel of the nursery. If arterial oxygen saturation (SPO_2_) was ≥ 95%, it was considered normal (first phase); but if it was < 95%, it was rechecked after 2 hours (second phase); and if SPO_2_ persisted to remain < 95%, it was considered abnormal and the newborn was presented to a single board-certified pediatric cardiologist for echocardiography and more detailed examination on the same day. Pulse oximetry was performed with a Nellcor NPB-295 pulse oximeter using a neonatal probe (DS-100A oximeter, Puritan-Bennett, Pleasanton, CA, USA). This device measured functional O_2_ saturation.

For the statistical analyses, the statistical software SPSS version 18.0 for Windows (SPSS Inc., Chicago, IL) was used. The discrete variables are expressed as counts (percentages) and the continuous variables as means ± standard deviations. The continuous variables were compared using the Student *t*-test, and the chi-square test or the Fisher exact test was used for the categorical data. A p value < 0.05 was considered significant.

## Results

**Figure 1:**
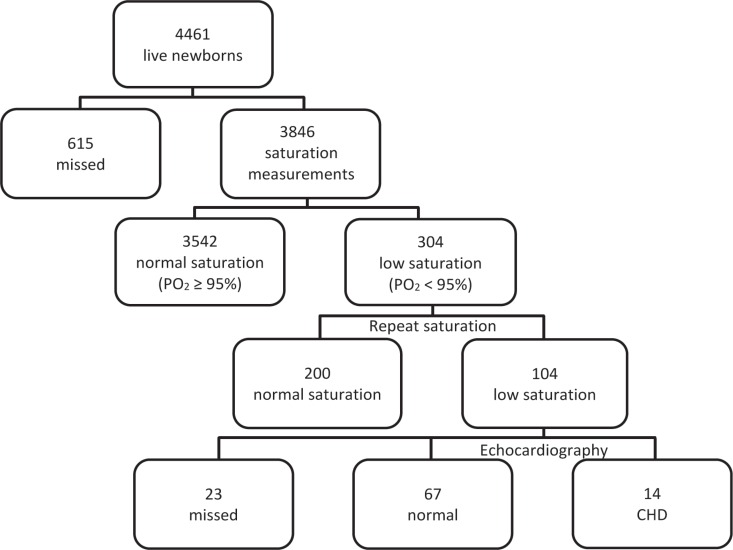
Flow chart of the study

CHD, Congenital heart disease; PO_2_, Oxygen saturation

During the study period, totally 4,461 neonates were delivered in Kashan Shabihkhani Maternity Hospital. A total of 3,846 newborns met the eligibility criteria for pulse oximetry screening. The mean gestational age, birth weight, and mother’s age of these eligible newborns were 38.52 ± 0.69 weeks (range = 38-41), 3313.6 ± 457.21 grams (range = 1250-5500), and 25.4 ± 6.1 years (range = 14-48), respectively. Of these neonates, 304 (7.9%) had SPO_2 _< 95% in the first phase. The second saturation measurement was performed 2 hours later, and 104 (2.7%) neonates had persistent low saturation. Figure 1 depicts the flow chart of the study design. The demographic data of the patients with SPO_2 _< 95% are illustrated in Table 1.

Echocardiography was performed on 81 out of 104 newborns, and the results demonstrated that 14 (0.36%) of them had CHD (11 cases of critical CHD and 3 cases of noncritical CHD) and the remaining 67 (1.74%) babies were healthy newborns with prolonged transitional circulation. From the 104 newborns who had abnormal SPO_2_ values (SPO_2 _< 95%), the degree of hypoxia was determined and divided into 2 groups: 75-84% and 85-94%. There was no difference in the severity of hypoxia between the 2 groups in terms of sex (p value = 0.587), delivery type (p value = 1), timing of pulse oximetry (p value = 0.305), and birth weight (p value = 0.379), but there was a significant difference between the 2 groups regarding the echocardiographic results (p value = 0.004) (Table 2). 

The timing of pulse oximetry was < 12 hours after birth in 25 (24%) neonates, 12-24 hours after birth in 60 (57.7%), and > 24 hours after birth in 19 (18.3%). The mean age of the neonates at the time of pulse oximetry was 18.91 ± 8.61 (min = 4.5 and max = 49) hours. In 13 patients with the diagnosis of CHD, pulse oximetry was performed before 24 hours of birth and just in 1 case screening was done after 24 hours (7.1%). On the other hand, most of the CHD cases in our study were diagnosed within 8-24 hours of life by first-day pulse oximetry screening. There was a significant difference in the diagnosis of CHD according to the timing of pulse oximetry (p value = 0.044) (Table 3). 

The types and characteristics of the CHD cases are shown in Table 4. 

**Table 1:** Characteristic of the patients with SPO_2_ < 95%

	Number	Mean	Standard Deviation	Min	Max
Weight (g)	104	3370.19	463.12	2400	4750
Head circumference (cm)	104	34.72	1.21	31.5	38
Height (cm)	104	50.71	2.11	46	56
Mother’s age (y)	104	26.6	5.28	16	39
Gravidity	104	2.00	1.11	1	7
Newborn’s age (h)	104	18.91	8.61	4.5	49
Heart rate (beat per min)	104	128	13.00	82	164
SPO_2 _(%)	104	89.87	4.36	75	94

SPO_2_, Oxygen saturation

**Table 2:** Characteristics of the newborns evaluated for hypoxemia in the patients with SPO_2 _< 95%

	75% ≤SPO_2 _≤ 84%	85% ≤ SPO_2 _≤ 94%	P value
Sex			0.587
Female	5	46	
Male	7	46	
Delivery			1
NVD	7	57	
C/S	5	35	
Timing			0.305
< 12 hours	5	20	
12-24 hours	5	55	
> 24 hours	2	17	
Weight			0.379
< 2500 grams	0	2	
2501-4000 grams	12	79	
> 4000 grams	0	11	
Echocardiography			0.004
Normal	6	61	
CHD	6	8	

SPO_2_, Oxygen saturation; NVD, Natural vaginal delivery; C/S, Cesarean section; CHD, Congenital heart disease

**Table 3:** Characteristics of the newborns evaluated by echocardiography in the patients with SPO_2 _< 95%

	CHD	Normal	P value
Sex			0.721
Female	8	33	
Male	6	34	
Delivery			0.224
NVD	10	36	
C/S	4	31	
Timing			0.044
< 12 hours	7	13	
13-24 hours	6	39	
> 24 hours	1	15	

SPO_2_, Oxygen saturation; CHD, Congenital heart disease; NVD, Natural vaginal delivery; C/S, Cesarean section

**Table 4:** Patients characteristics and SPO _2_ in the neonates with CHD*

Number	Sex	Age (hr)	SPO_2_ (second phase-2 hours later)	Disease Type
1	Male	26	82	VSD + PH
2	Male	14	77	ASD + PH
3	Male	9	82	PPHN
4	Male	21	87	DORV + VSD + PS
5	Female	8	91	SV + TGA
6	Female	9	87	VSD + PA
7	Female	15	93	TOF
8	Female	10	94	TA
9	Male	18	92	TOF
10	Female	16	94	TOF
11	Female	16	84	TAPVR
12	Male	10	75	TGA + IVS
13	Female	12	85	DORV + VSD + TGA
14	Female	12	80	AVSD + PA

SPO_2_, Arterial oxygen saturation; TOF, Tetralogy of Fallot; TGA, Transposition of the great arteries; TAPVR, Total anomalous pulmonary venous return; VSD, Ventricular septal defect; ASD, Atrial septal defect; PH, Pulmonary hypertension; PPHN, Primary pulmonary hypertension; DORV, Double-outlet right ventricle; TA, Truncus arteriosus; PA, Pulmonary atresia; IVS, Intact ventricular septum

## Discussion

Timely diagnosis of critical CHD in neonates, before the onset of symptoms, is an important clinical challenge. The screening for critical CHD previously relied on prenatal ultrasound and physical examination after birth; both, however, had abilities to detect critical CHD. As the most neonates with critical CHD have a degree of hypoxemia, attention has more recently focused on pulse oximetry as a screening method for the early detection of critical CHD.^11^

Our study allowed early diagnosis of CHD in 14 neonates, including 11 neonates with critical CHD who were all asymptomatic. Most of these detected CHD cases might have been life-threatening if left undiagnosed. The present study showed specificity and sensitivity of 98.3% and 61%, respectively. Some studies have shown that pulse oximetry screening has high sensitivity and specificity for detecting severe CHD in apparently healthy neonates.^12^^, ^^13^ A recent systematic review and meta-analysis of 13 studies including 229,421 neonates revealed that the sensitivity and specificity of pulse oximetry was 76.5% and 99.9%, correspondingly. According to these findings, pulse oximetry fulfill the criteria for universal screening because it is highly specific and moderately sensitive for the detection of critical CHD.^11^^, ^^14^

False positive results arouse in 0.8% of our cases, which is consistent with the results from the PulseOx Study in the United Kingdom.^15^ These results were due to pulmonary hypertension. Early detection of persistent pulmonary hypertension is an important additional advantage of this test because it can draw attention to a serious noncardiac problem (early onset sepsis or a respiratory problem) before the baby becomes sick. About 30-70% of the false positive tests fell into this category according to recent studies.^11^^, ^^13^ Early detection of pulmonary diseases and infection problems may represent a secondary target for pulse oximetry. There is no doubt that screening after 24 hours has a lower false negative rates; nonetheless, as there is a tendency to discharge apparently healthy newborns before 24 hours, waiting until after 24 hours to perform a screening test is impracticable. In our study, the timing of pulse oximetry was < 12 hours after birth in 24% of the neonates, 12-24 hours after birth in 57.7%, and > 24 hours after birth in 18.3%. Most of the cases were diagnosed before 24 hours of life. The mean age of the neonates at the time of pulse oximetry was 18.91 ± 8.61 hours. In a study by Richmond,^7^ the mean age at pulse oximetry was 11.7 hours, which is almost the same as that in our study. The mean age at pulse oximetry in a study by Arlettaz^16^ was 8 hours. The average age at pulse oximetry screening was 7.3 hours in a Polish study, and it was associated with a low false positive rate.^17 ^The timing of pulse oximetry in a study by Hoke^9^ was < 24 hours in 37 newborns, after the first day of life in 8, and at discharge in 12. In a study by Baker,^18^ the mean age at pulse oximetry was 31.7 hours. Koppel et al.^6^ conducted screening after 24 hours of birth and much closer to discharge with a very low false positive rate. A balance should exist between the timely diagnosis of life-threatening situations and an excess of false positive rates. If pulse oximetry is done a few days after birth, the number of false positive results will be reduced due to a reduction in pulmonary vasculature resistance. Nevertheless, CHD patients with ductal-dependent pulmonary circulation will experience fatal events in this period. Pulse oximetry very soon after birth will increase the number of undue echocardiographic examinations. However, this can increase the diagnosis rate of CHD with a poor prognosis and delayed intervention. We found that a high false positive rate of pulse oximetry due to high pulmonary artery pressure prompted more detailed clinical examination and echocardiography, which is worth performing in critical patients. The AAP recommends that all asymptomatic neonates be screened for critical CHD between 24 and 48 hours of age or prior to discharge.^19^

In addition to the timing of the screening, the cutoff value and the position of the pulse oximetry probe can influence the accuracy of screening. A higher threshold could increase sensitivity but decrease specificity, and a lower threshold will decrease sensitivity but increase specificity. Different researchers have used various cutoff values for the lower limit of normal saturation between 92 and 96%, but most of them have considered SPO_2_ < 95% as an appropriate threshold.^6^^, ^^8^^, ^^15^^, ^^16^ Although many studies have measured postductal saturation on either foot,^6^^, ^^7^ a meta-analysis demonstrated no difference in sensitivity between postductal testing and preductal testing combined.^14^ Still, it is more accurate to measure both preductal and postductal saturations (with a difference > 3% considered abnormal), which may improve the detection of the rare cases of the transposition of the great vessels with a large patent ductus arteriosus.^9^^, ^^15^ The simplicity of postductal measurement is an advantage for the purpose of large-scale screening.^19^

The major limitation of our study is that not all the neonates with abnormal screening underwent echocardiography. In addition, clinical follow-up was not available for some of the infants, and no cases of coarctation of the aorta were detected by our study. Accordingly, our results can express only the positive predictive value of pulse oximetry. 

Despite the ability of pulse oximetry to detect most cases of critical CHD, it is not without limitation. The commonest lesions missed by pulse oximetry are those causing obstruction to the aorta (e.g., coarctation and interrupted aortic arch); they are even frequently missed by prenatal ultrasound and routine physical examination.^11^^, ^^20^ Hospital staff and parents should be informed about these limitations. Therefore, pulse oximetry may be used as an adjunct to other screening methods rather than as a substitute.

## Conclusion

Pulse oximetry is a safe, noninvasive, cost-effective, and feasible screening tool adjunct to standard physical examination of neonates for the detection of critical CHD in apparently healthy newborns and may become a part of discharge plan for every newborn.

## Notes:

***This paper should be cited as:*** *Movahedian AH, Mosayebi Z, Sagheb S. Evaluation of Pulse Oximetry in the Early Detection of Cyanotic Congenital Heart Disease in Newborns. J Teh Univ Heart Ctr 2016;11(2):73-78.*
